# The role of herpesvirus 6A and 6B in multiple sclerosis and epilepsy

**DOI:** 10.1111/sji.12984

**Published:** 2020-10-23

**Authors:** Nicky Dunn, Nastya Kharlamova, Anna Fogdell‐Hahn

**Affiliations:** ^1^ Department of Clinical Neuroscience Karolinska Institutet Stockholm Sweden; ^2^ Center for Molecular Medicine Stockholm Sweden

**Keywords:** autoimmunity, epigenetic modifications, epilepsy, multiple sclerosis, viral infection

## Abstract

Human herpesvirus 6A (HHV‐6A) and 6B (HHV‐6B) are two closely related viruses that can infect cells of the central nervous system (CNS). The similarities between these viruses have made it difficult to separate them on serological level. The broad term HHV‐6 remains when referring to studies where the two species were not distinguished, and as such, the seroprevalence is over 90% in the adult population. HHV‐6B has been detected in up to 100% of infants with the primary infection roseola infantum, but less is known about the primary infection of HHV‐6A. Both viruses are neurotropic and have capacity to establish lifelong latency in cells of the central nervous system, with potential to reactivate and cause complications later in life. HHV‐6A infection has been associated with an increased risk of multiple sclerosis (MS), whereas HHV‐6B is indicated to be involved in pathogenesis of epilepsy. These two associations show how neurological diseases might be caused by viral infections, but as suggested here, through completely different molecular mechanisms, in an autoimmune disease, such as MS, by triggering an overreaction of the immune system and in epilepsy by hampering internal cellular functions when the immune system fails to eliminate the virus. Understanding the viral mechanisms of primary infection and reactivation and their spectrum of associated symptoms will aid our ability to diagnose, treat and prevent these severe and chronic diseases. This review explores the role of HHV‐6A and HHV‐6B specifically in MS and epilepsy, the evidence to date and the future directions of this field.

## 1 INTRODUCTION

Human herpesviruses (HHV) 6A and 6B (HHV‐6B) are large, enveloped double‐stranded DNA betaherpesviruses. They are considered as ubiquitous in the population but have been notoriously hard to distinguish from each other with serological assays, and therefore, the precise view of the geographical and age distributions of these two viruses is yet to be determined. Here, we will refer to them as HHV‐6 when citing studies where they have not been separated, and as such, they occur in over 90% of the adult population and establish itself early in life.^1^ Primary infection with HHV‐6B generally occurs in infancy, when the protective maternal antibodies wane, and presents with a self‐limiting undifferentiated febrile illness and in a portion with roseola infantum.^2^, ^3^, ^4^ Although there are less epidemiological data about HHV‐6A, it seems to be more common in Africa and to have an asymptomatic primary infection.^5^ Similar to other herpesviruses, HHV‐6 viruses are extremely efficient at evading the immune system, and as a result, they can establish lifelong latency in the host in wide range of cell types including cells of the central nervous system (CNS). Viral reactivation of HHV‐6 can occur, particularly in immunosuppressed hosts. This can lead to a number of diseases including acute encephalitis, but has also been implicated in the pathogenesis of chronic neurological diseases such as multiple sclerosis (MS) and epilepsy.^6^, ^7^

There are several ways that viruses are implicated in pathogenesis of chronic brain diseases. In animal models, the same virus (Theiler's murine encephalomyelitis virus) can cause either MS‐like disease or epilepsy depending on which mouse strain (SJL/J or C57BL/6J) is infected intrathecally.^8^ This indicates the importance of host genetics in regulating immunity and pathogenesis. HHV‐6B has been detected in brain tissue surgically removed from patients with epilepsy.^7^, ^9^, ^10^ Persons with MS have an increased serological response to a selected antigen from HHV‐6A, but not from HHV‐6B, and this increase was also seen in serum collected before the MS diagnosis and thus it confers a risk factor for MS.^11^ In addition, HHV‐6A and HHV‐6B are implicated in a number of other neurological diseases which commonly present with seizures or encephalitis, including HHV‐6 post‐transplant acute limbic encephalitis (HHV‐6 PALE), Rasmussen encephalitis and febrile seizures in children.^12^ It is important to consider these complications of HHV‐6 infection in patients treated with immunomodulatory drugs due to their associated risk of treatment‐related opportunistic infections.^13^ This review will, however, only explore the role of HHV‐6A and HHV‐6B specifically in epilepsy and MS, the evidence to date and the future directions of this field.

## 2 MAIN TEXT

### 2.1 Human herpesvirus 6A and 6B structures and immune system interactions

**FIGURE 1:**
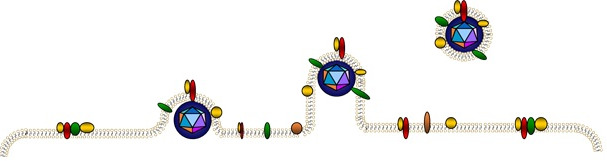
The concept of how larger enveloped viruses form their membrane by budding out of the host cell membrane and thus bringing the proteins and lipids from the host cell integrated into the viral particle

There are nine herpesviruses known to be human pathogens. HHV‐6A and HHV‐6B are two distinct species belonging to the β‐herpesviruses subfamily in the roseola virus genus. Despite sharing 90% of their nucleotide sequence, the two virus isolates demonstrate both genetic and phenotypic variation.^14^, ^15^ They were initially classified as separate strains of the same virus, but were in 2012 concluded to be two distinct species.^16^ Through epigenetic modifications and histone modification, the virus can integrate itself into the telomeric part of the chromosomes of infected cells.^17^, ^18^ This includes the germ cells, from where it can be inherited in a mendelian fashion, resulting in 1% of the human population having HHV‐6A or HHV‐6B genome integrated into the genome of all cells in the body.^19^ One defined receptor for HHV‐6 infection is the complement inhibitor CD46.^20^ It is a cell surface protein expressed on all nucleated cells and thus gives the viruses a very large range of possible host cells, including CNS cells such as oligodendrocytes, astrocytes and glial cells.^21^ This receptor is considered to be more exploited by HHV‐6A, whereas HHV‐6B uses CD134, a primarily T cell surface protein, as a primary receptor. HHV‐6B might then have a more restricted tropism that would not include the brain cells.^22^ However, large and enveloped viruses such as human herpesviruses can incorporate cellular proteins from the host cell into its envelope, including their own receptor.^23^ Therefore, for every new cell type the virus infects and replicates from it will change its protein content and acquire a new set of potential receptors and thereby possibly expand its tropism (Figure 1). This mechanism of incorporation of in particular complement inhibitory proteins could be a general immune evasion strategy of viruses. Several viruses are known to have the complement inhibitory proteins CD46, CD55 and CD59 incorporated in their envelope, which will protect them against complement lysis.^24^, ^25^, ^26^ Incorporation of host cells proteins and lipids into larger enveloped viral particles is a well‐known phenomenon by virologists, but deserves to be explored further by immunologists to determine which immunopathological events might be explained by this mechanism.

The proteins that are incorporated following infection of brain cells have not yet been investigated, and we do not know if this could lead to autoimmunity. In autoimmunity, molecular mimicry is a widely accepted hypothesis, postulating that expression of microbe‐encoded genes generates protein and peptide epitopes which are sufficiently similar to mammalian host‐encoded proteins to evoke cross‐reactive activation of the immune system.^27^ In contrast to the incorporation theory, the mimicking sequence triggering autoreactive T and B cells can be identified on nucleic acid level, by comparing the host genome to the viral genome. Incorporated antigens, however, can only be identified using proteomic analyses of viral particles cultivated in the host cell of interest. Since viral incorporation adds a new dimension to the molecular mimicry hypothesis, it is intriguing to explore how the immune system will handle viral particles with different cellular protein and lipid components and if cell‐specific autoimmunity is triggered dependent on which cell type the virus is propagated in.

### 2.2 The role of human herpesvirus 6A in multiple sclerosis

Many autoimmune diseases have been associated with different herpesviruses (reviewed in Ref. (28)). MS is a chronic, inflammatory, demyelinating disease of the central nervous system. The aetiology of MS is unknown, but both genes and environmental risk factors have been identified.^29^ Several different viruses have been suggested to be associated with MS, but what makes HHV‐6 an attractive candidate for MS aetiology is that it has been found to be present in the oligodendrocytes of MS plaques.^30^, ^31^

Several studies have investigated the specific association between HHV‐6 and MS.^6^, ^32^, ^33^ There are contradictory results, but a role for HHV‐6A has been proposed^34^, ^35^, ^36^ and an association with circulating IgM levels with MS has been replicated.^32^, ^37^ The difficulty to distinguish between HHV‐6A and HHV‐6B with serological methods might be one explanation for the discrepancies of these studies. However, in a collaborative project investigating the serological response of over 8500 persons with MS and around 7000 healthy controls, as well as in samples taken before disease onset, we could recently support an association between seropositivity against the HHV‐6A antigen IE1A (OR = 1.55, *P* = 9 × 10^−22^) and increased risk of future MS (OR = 2.22, *P* = 2 × 10^−5^), but not for the corresponding HHV‐6B antigen IE1B.^11^

Since as described, HHV‐6A is a large enveloped virus and can incorporate host cell proteins into its envelope (Figure 1), it would be an attractive candidate for the alternative mechanism of incorporation, worth exploring as an aetiological factor of autoimmunity in MS.^23^, ^38^ For MS, viral particles propagated in the myelin‐producing oligodendrocytes might contain myelin proteins/lipids, but this has not been characterized yet. There are, however, several interesting examples from other enveloped viruses, indicating that this is a general phenomenon, such as human Immunodeficiency virus (HIV) which incorporates several proteins,^39^, ^40^ and vesicular stomatitis virus (VSV) which incorporates myelin basic protein (MBP) when cultivated in MBP‐expressing cells.^41^ When encountered by the immune system, antigen‐presenting cells (APCs) that have phagocytosed these viruses would present a wide range of peptides, derived from both viral proteins and incorporated myelin proteins from the oligodendrocyte host cell. Hence, specific B and T cell response against antigens from both the virus and the host cell might be activated. In addition, lipid‐specific B cells might become activated against the lipids of the host cell when these are taken up as part of the viral envelope by the B cell. One other virus with known tropism for oligodendrocytes, the John Cunningham virus (JCV), is a less likely candidate for autoimmunity since it is non‐enveloped and causes rapid viral‐mediated fatal disease of the host. Therefore, this pathology would be completely different from the incorporation hypothesis for autoimmunity, as we suggest is also the case for the role of HHV‐6B in epilepsy.

### 2.3 The role of human herpesvirus 6B in epilepsy

Epilepsy is a common disease characterized by the uncontrolled overexcitatory activity of neurons, which results in seizures and convulsions. There are several different causes of the disease, but in many cases, the aetiology is unknown. To date, there are no cures for epilepsy, but the seizures are controlled by anti‐epileptic drugs. In severe medically refractory epilepsy, surgery is used to remove the part of the brain from where the seizures originate.

In surgically excised hippocampal tissue from patients with mesial temporal lobe epilepsy (MTLE), we and others have found high HHV‐6B viral DNA load,^7^, ^10^ indicating an association between the virus and epilepsy and indicating that HHV‐6B infection can be found in the brain despite the lack of expression of CD134 (according to the protein atlas).^42^ In the epileptic tissue, HHV‐6B viral proteins were detected in astrocytes, indicating at least partial activity of the virus through gene expression.^7^, ^43^ These cells are important for the clearing of neurotransmitters to terminate neuron signalling, and the presence of viral proteins, even without complete viral replication, might hamper this function.

Using a nested PCR technique, HHV‐6 has been recorded in 24%‐43% of brain biopsies collected from healthy individuals.^44^, ^45^, ^46^ However, even if viral DNA is present, viral proteins do not always seem to be expressed.^46^ In vitro studies have shown that human glial precursor cells can be infected by both HHV‐6A and HHV‐6B,^47^ while only HHV‐6A establishes a productive infection in astrocyte cultures.^48^, ^49^ HHV‐6B does not replicate in astrocytes in vitro, but low levels of DNA can be detected in the cells for >6 cell passages,^49^, ^50^ suggesting that HHV‐6B might become latent in astrocytes.

One hypothesis is that HHV‐6B establishes itself as an infection in the brain during childhood in glial precursor cells, possibly only as incompletely replicating, but enough to cause modifications of the host cells. Alternatively, latency could be followed by recurrent incomplete reactivations, enough to hamper the function of the astrocytes to a degree that affects the clearance of neurotransmitters, thus causing epileptic symptoms.

It has been suggested that latency of herpesviruses is regulated by epigenetic mechanisms. Primarily, upon virus entry of a cell, the viral genome is folded around histones and the ‘tails’ are modified to repress or enhance gene expression (reviewed in Ref. (51)). Viral DNA also becomes methylated, which is a marker of transcriptional repression and possibly an effort of the host cell to control the virus. Quiescent cytomegalovirus (CMV), a herpesvirus closely related to HHV‐6, reactivates from latency upon de‐methylation of the viral DNA and by de‐acetylation of histone tails.^52^ We have discovered that HHV‐6B affects the host cell subtelomere methylation during active infection, which correlates with virus integration into the host cell telomeres, indicating that the virus uses the host epigenetic machinery to modify the epigenetic landscape for its own survival.^53^ In recent years, it has been suggested that epigenetic modulations might play a role in epilepsy,^54^ but these studies did not investigate the presence of the virus in the tissue.

Our preliminary results indicate that HHV‐6B affects the MAPK kinase signalling pathway, both in vivo of brain tissue from epilepsy brain surgery and in vitro in T cell lines. This pathway has been shown to be affected in status epilepticus^55^ and that some proteins in this pathway are activated in areas with astrogliosis in epileptic hippocampal tissue.^56^ More specifically, activation of the MAPK cascade can trigger local energy deficit^57^ and cytoskeleton reorganization during viral infection.^58^ It has been shown that MAP2K4/MKK4 is important for induced vacuolization of glioblastoma cells,^59^ which might be associated with the two classical features of HHV‐6B infection that are change in cell morphology and vacuolization of cells. However, the molecular mechanisms underlying these morphological changes if they are important for vesicle transportation have not as yet been investigated. Thus, HHV‐6B might contribute to the pathogenesis in epilepsy by interfering with the vesicular transport system through the MAPK pathway, leading to disturbed glutamate uptake by infected astrocytes. Both viral‐induced epigenetic modification and how HHV‐6B modifies host cell functions deserve further investigations, and possible mechanisms of how this might lead to epileptic symptoms are of interest to explore. The challenge for immunologist here is to understand how the viral infection can establish itself in the brain without any apparent detection and elimination efforts by the immune system.

## 3 SIGNIFICANCE AND FUTURE PERSPECTIVE

We are only at the start of understanding of the significance of HHV‐6A and HHV‐6B in MS and epilepsy and the contrasting ways they may cause pathology. Future studies have the potential to contribute in enhancing the basic knowledge about these viruses in different cell types, improving the sensitivity, specificity and throughput of diagnostic tests, as well as finding suitable treatments, interventions and potential prophylaxis.

Basic scientific knowledge about how HHV‐6A and HHV‐6B efficiently evade the immune system and, similar to other herpesviruses, establish latency with potential to reactivate later in life needs further investigations. By increasing our understanding of the molecular and cellular mechanisms driving these processes, we not only learn more about specific neurological diseases, but also about the immune system regulations and viral infections in general and the counteracting immune evasion strategies. To address whether incorporation of host cell proteins and lipids in the virion might be an alternative mechanism to explain the specificity of autoimmunity in general, the virus needs to be cultivated in the target cell of the disease. Even though incorporation is not new in the virology field, we are only in the beginning of understanding how the immune system handles these host cell‐specific viral particles. For MS, characterizing a significant host cell‐specific protein signature of HHV‐6A from human oligodendrocytes should be prioritized. At the other end of the spectrum, the ability of HHV‐6B to hamper the function of human astrocytes, without evoking extensive immune reactions, should be studied in more detail including epigenetic modifications; chromosomal integrations; and potential reactivation strategies targeting these viral‐induced modifications. With recent advancements making it possible to cultivate human CNS cells in vitro as organoids from inducible pluripotent stem cells, single‐cell analysis and high‐throughput ‘omics’ technology platforms, the feasibility of these types of experiments has increased. An alternative is to investigate this mechanism in the marmosets, an animal model that develops MS‐like symptoms when infected with HHV‐6A but not with HHV‐6B.^60^ Further explorations of other viruses with similar characteristics and tropisms as HHV‐6A and HHV‐6B, which might also stimulate similar autoimmune responses or viral‐mediated dysfunctionalities, are warranted.

Diagnostic testing of HHV‐6A and HHV‐6B is centred around detection of DNA with PCR or antibodies with serology. The primary limitation of identifying an active infection by detecting extracellular DNA with PCR test is that it will only be positive if your sample is taken during a viremia, which typically only lasts a few days after infection, and thus, the time window to detect the virus is very narrow. Although this method is useful for triaging patients with severe acute infections in the hospital, it will be less informative as a potential diagnostic tool for chronic diseases such as MS and epilepsy. Here, one would have to apply a combination of methods detecting HHV‐6A and HHV‐6B DNA and proteins in cerebrospinal fluid compared to sera in conjunction with specific serological tests of IgM and IgG, as has been done in studies of febrile seizures and epilepsy.^61^ Clearly, since almost everyone is infected, it is not enough to detect the virus or immune responses to it, but the additional events that might lead to CNS pathologies need to be characterized and used as diagnostic tools. This is particularly true for epilepsy, where no viral DNA was detected in blood, although high copy numbers were detected in hippocampus of the same individuals.^7^ Similarly, no viral DNA or pleocytosis was detected in CSF in the children with febrile seizures associated with HHV‐6B.^61^ Thus, to be able to identify cases where HHV‐6B is present in astrocytes and is causing pathology, novel inductions of biomarkers or downregulation of essential housekeeping genes might be alternatives worth exploring.

Discoveries of novel biomarkers in CNS cells from in vitro cultures could potentially enable identification of these viral traces in clinical samples. The ability of the viruses to integrate their genome early in acute infection of neural cells through epigenetically regulated mechanisms, affecting gene expression and inducing dysregulation of neural host cell functions, should be explored further. In the case of the incorporation hypothesis for autoimmunity, a combination of serological specificities both against the virus and the potentially incorporated host cell proteins from human oligodendrocytes might reveal if such events happen in vivo and are of clinical relevance. These could be used to better stratify patient groups, and if these viruses have any implications for the pathogenesis of CNS diseases, diagnostic tests for characterizing subgroups of epilepsy and MS can be developed. These would enable identification of when the viral infection should be treated.

Development of therapeutic interventions for HHV‐6A and HHV‐6B infection, reactivation and complications is centred around antiviral therapy, where a limited set of drugs is available. Patients with complicated HHV‐6 infection are treated with antiviral drugs such as ganciclovir despite being toxic and in some cases ineffective against HHV‐6 infection.^62^ Upcoming novel alternatives are for example the recent advances in immunotherapies including viral‐specific adoptive T cell immunotherapy and HHV‐6‐specific neutralizing antibodies that have been successful in early trials.^63^, ^64^ Furthermore, strategies using CRISPR‐Cas9 to target viral genomes are also in progress.^65^ This will be imperative for patients with MS or epilepsy to remove the virus from the specific target cells, if found to be contributing to disease activity and progression. Thus, a better understanding of specific pathogenic mechanisms will enable development of new treatments strategies directed against the pathways dysregulated by the virus, epigenetic modifications or the integrated DNA of the virus. Potential future prophylaxis is currently developed for HHV‐6 and other viruses, using T cell infusions.^66^ However, given the ubiquitous nature of these viruses, the possible negative implications of eradication of HHV‐6A and HHV‐6B must be considered when deciding on the use of prophylactics. It has been suggested that herpesviruses might form a symbiotic relationship with the host with their systemic activation of macrophages and induction of the interferon system, protecting the host against lethal bacterial infections.^67^ Whether this protection is true for humans and for how long it might last remains to be determined, but it indicates that removing highly adapted viral infections from the human population might have unforeseen consequences.

## CONFLICT OF INTEREST

The authors have no conflict of interest to report.

## AUTHOR CONTRIBUTIONS

AFH drafted the manuscript. ND and NK reviewed, edited and approved the final version.
